# On the Influence of Climate in the Prevention and Cure of Chronic Diseases, More Especially of the Chest and Digestive Organs, &c.

**Published:** 1830-01-01

**Authors:** 


					IX.
The Influence of Climatic in the Prevention and Cuke
of Chronic Diseases, more especially of the Chest and
Digestive Organs, Sec.
By James Clark, M.D.
LArt. II. Conclusion.]
Consumption.
In a former article we gave a full account of the first part of Dr. Clark's
Work on Climate. The second part of the same is chiefly occupied with
observations on disorders of the thoracic and abdominal viscera. Dr.
Clark's long residence in Italy, where he had such extensive opportunities
of witnessing not only the effects of climate on pulmonary diseases, but
also the phenomena of these diseases themselves, in all their stages and
varieties, gives a great weight to his opinions, and attaches much importance
to his observations. On this account we shall pass over the section on
disorders of the digestive organs, because the subject is almost exhausted,
and because Dr. Clark is probably more at home on the subject of pulmo-
nary affections, for which, rather than for dyspeptic complaints, our invalids
seek the blue skies and mild air of Italy.
Dr. C. commences with a sentence gloomy enough, but perhaps not
uncongenial with the subject.
" There is no disease in which change of climate is considered in this country
of so much importance, as Consumption ; and yet it must be admitted, that
there is no one in which the hopes founded in such a change, are more con-
stantly disappointed. Occasional examples of its beneficial effects are certainly
observed; and, though they bear so trifling a proportion to the cases in which
it produces no benefit, or only a temporary and very trifling one, it still remains
the chief source of hope and confidence in the treatment of consumption, both
to the profession and the public." 223.
A belief in the efficacy of change of air in this terrible malady is of
ancient date, and is still universal over the civilized world. In this country,
where consumption is so prevalent, the above-mentiened belief is more
strong than in most other regions ; and the failures are attributed to the
late period at which the change of air is made, rather than to the inefficacy
of the measure.
" For my own part, although I have witnessed the melancholy issue of too
many cases sent abroad at a very early period of the disease, to have much
confidence in such a measure singly, still 1 believe a change to a mild climate,
or rather a temporary residence there, to be a valuable remedy, under certain
limitations. And 1 am further of opinion, that when a more rational view of the
nature and causes of consumption is generally adopted, change of climate may
1829] Dr. Clark on Climate in Consumption. 91
be made a far more efficient and a more certain remedial agent in that disease,
than it has hitherto been." 224.
Dr. C. thinks that, notwithstanding the frequency of consumption here,
and the numerous works that have been published respecting its causes,
nature, and treatment, yet that these are very imperfectly understood, while
the faculty themselves are divided in opinion about them. In respect to
treatment, he thinks there is no reason to believe that the physicians of the
present day are more successful than their predecessors were ten, nay
twenty centuries ago !
Taking tubercles as a fixed point from which to start in his investigations,
Dr. C. observes that, " until we arrive at a knowledge of the state of the
system which leads to the formation of these bodies, and of the causes
which induce this state, we cannot hope to establish rules for the prevention
of consumption upon any sound principle." Again. " I say prevention
of consumption?because to cure it, even in its earlier stages, in other words,
to remove tubercles already existing in the lungs, is what we can scarcely
hope to do." Laennec indeed has remarked that " the cure of tubercular
consumption is not beyond the powers of Nature?but art has not been
found equal to the task." The utmost which we can expect is to retard the
progress of tubercles, when once they are formed. The expulsion from
the lungs of tuberculous matter occasionally leads to a permanent cure, and
this, we fear, is the only one.
Dr. C. adverts to the litigated question, " are tubercles the product of in-
flammation, or are they the result of a specific action," totally unconnected with
phlogosis, arising out of a morbid condition of the general system ? Much
of the preventive treatment depends on the solution of this question. Our
author, like most of the modern pathologists believes that the formation
and growth of tubercles are not essentially connected with inflammation.
" It is obvious that, in order that tubercles should be the result of inflam-
mation, there must be some modified condition of the inflammation ; and if we
inquire more closely we shall find that another disorder is connected with the
inflammation, and that this disorder is the essential agent in the production of
tubercles. In a healthy subject, I believe tubercles are never the result of in-
flammation. When, therefore, these appear to be the product of inflammation,
it will be found to be inflammation occurring in and modified by a disordered
state of the system of a peculiar kind. And, surely, it is more reasonable to
attribute the tubercles to this cachectic state, which is almost constantly ob-
served, than to the inflammation which is only occasionally detected, and which,
in innumerable instances, is found to be of itself insufficient for their pro-
duction." 231.
Dr. C. does not mean to underrate the injurious effects of pulmonary
inflammation in persons disposed to consumption, or already labouring
under that disease in its incipient stages. On the contrary he is aware of
its importance, and of the necessity of speedily removing it, as he believes
that inflammation accelerates the progress of phthisis. Our author has
drawn a light but correct sketch of the symptoms which denote the early
progress of pulmonary tubercles, which he denominates tubercular cachexy,
and which is preceded by a deranged state of the constitution, and followed
by the actual disease itself.
"Under the general term, consumption, then, we may comprehend three
different forms or stages of disease?1st, general disorder of the health ; 2nd,
tubercular cachexy ; 3rd, consumption, properly so called." 242.
92 MKUJCo-ciiinuitGicAL Review. [Nov.
. Of the causes predisposing, hereditary, and exciting, wo need not speak,
as they are sufficiently discussed in various publications.
As it is useless to talk of curing consumption, our only hope is to prevent
its occurrence in those who are predisposed to it. The disorder of 'the ge-
neral health being attended to, a change of air to a milder climate may be of
some service in preventing or retarding the progress of the complaint. The
profession is now aware of the impolicy?the cruelty of sending patients to
a warm climate after tubercles have begun to soften down and to be ex-
pectorated. Still there are annual instances of people being sent, by thought-
less friends and physicians, in this state, to die in Italy, France, or on the
road.
" When removal to a mild climate is decided on, the next subject which na-
turally presents itself for consideration, regards the selection of that which is
most suitable to the case. The question has been often put to me?Which is
the best climate ? The truth is, no one climate or situation is the best in all
cases. In the first part of this work I have given the character of the climate
of the different places resorted to by invalids, and have endeavoured to draw a
comparative view of their respective merits $ and to this I beg to refer the reader.
With regard to the climates of the south of France and Italy, I may here ob-
serve, that for consumptive invalids, in whom there exists much sensibility to
harsh and keen winds, and, more especially, if the immediate vicinity of the sea
is known to disagree, Rome or Pisa are the best situations for a winter residence.
When, on the contrary, the patient labours under a languid or oppressed cir-
culation, with a relaxed habit, and a disposition to congestion or to haemorrhage
rather than to inflammation, and, more especially, where the sea air is known by
experience to agree with the individual,?Nice deserves the preference. In
cases complicated with gastric irritation, however, Nice is an improper resi-
dence, its climate being decidedly inimical to this state. The climate of Hyeres
may be considered as similar to that of Nice in this respect. The influence of
such a morbid condition of stomach in modifying all other diseases, is sufficient
to claim for it the chief consideration in deciding upon the particular situation ;
although, I fear, it is but seldom thought of when the physician is deciding
which climate deserves to be preferred. Judging, however, from experience, I
should say, that where this state of the stomach exists, a climate which disa-
grees with it, will do the patient little good, whatever may be the other disease
under which he labours.
" With those cases of chronic consumption, therefore, to which I have alluded,
and which, according to my observation, are almost invariably complicated with,
and, I believe, in a large proportion of cases, chiefly induced by disorder of the
digestive organs, Nice will decidedly disagree ; and, besides the gastritic dys-
pepsia, such patients have generally an irritated state of the bronchial mem-
brane, with a dry state of the skin and a morbid degree of sensibility of the
nervous system,?in all of which states that place is unfavourable. Rome or
Pisa will agree better with this class of invalids.
" But the climate which of all others I consider the best suited to consump-
tive patients generally is that of Madeira. It will be seen by a reference to the
meteorological tables in the Appendix, and from the comparisons which l have
made between the climate of this island and that of the different climates on
the continent of Europe in the article on Madeira, that the winter temperature
is considerably higher and more equable, and the summer heat much more mo-
derate than at any of these places. To such consumptive patients, therefore,
as are likely to derive benefit from climate, I consider Madeira as affording alto^
gether the best residence. And this opinion does not rest merely on a consider-
ation of the physical qualities of this climate, but is warranted by the experience
of its effects on those cases of consumption which alone ought to be sent abroad,
as will be seen by a reference to Dr. Kenton's tabic. Madeira has also this
1829] Dr. Clark on Cr,i>YfAffi in Consumption. 93
advantage (a very great one in my opinion) over all the other places in the south
of Europe, that the patient may reside there during the whole year, and thus
avoid the inconveniences and even risks attending a long journey, to which con-
sumptive invalids who pass the winter in Italy must be exposed. The summer
climate of the whole Mediterranean, is unsuited to consumptive invalids, and
indeed is known by experience to be so pernicious to them, that sailors and
soldiers attacked with the disease in the Mediterranean fleet, and garrisons of
Malta, &c., are invariably sent to England on the approach of summer." 267-
Dr. Clark is decidedly of opinion that a sea voyage is beneficial in the
early stages of consumption, and especially when the disease is.accompanied
by hajmoptysis.
We can only make room for one short extract before we concludethis article.
" There is yet another measure in the treatment of consumption which re-
quires some notice in this work, as it has been recommended as a substitute for
change of climate. In place of sending consumptive patients to pass the winter
in a milder climate, it has been proposed to keep them in rooms artificially
heated and maintained at a regulated temperature. With the advocates of such
a measure the state of the lungs appears to be the only consideration ; but it
need not be told, that without improving the general health, which cannot be
done without exercise in the open air, all our measures directed to the local
disease will be fruitless. We may by such means keep down inflammatory ac-
tion in these organs, but we shall be favouring the very condition of the system
which led to the disease, and the removal of which condition can alone afford
the patient a hope of recovery. In the incipient stages of consumption, there-
fore, I consider such a measure as the most improper that can in general be
adopted. In the advanced stages of the disease, on the other hand, when all
hopes of recovery have vanished, and when removal to a distant climate is totally
useless, life may be prolonged, in many cases, by keeping the invalids in apart-
ments, the temperature of which is regulated in such a manner as to maintain
the air in as pure a state as may be. Females will, cceteris paribus, bear such
a system of confinement better than males, from the circumstance of its being
more congenial to their usual habits of life. Also in consumption and chronic
bronchial disease occurring in the more advanced periods of life, such a measure
promises to be much more frequently beneficial than in early life. In cases of
inflammation of the lungs also which have occurred during the winter, confining
the patient entirely to the house in a regulated temperature, till all symptoms of
the disease have ceased, and until the return of mild weather, will be very judi-
cious, more especially when such a person is hereditarily disposed to consump-
tion. But when a person so circumstanced has the means, he should pass the
following winter in a climate where confinement would be unnecessary, and
where he might improve his general health by exercise in the open air," 2/3.
We regret our inability to notice the short sections on chronic diseases of
the larynx, trachea, and bronchia?on asthma?gout?chronic rheumatism,
in which many judicious observations are to be found. But the greatest
merit of the work consists in the extraordinary labour, and we believe ac-
curacy, with which Dr. Clark has constructed a series of meteorological
tables, ten in number, from the best authorities, shewing the state of ther-
mometer, in every season, inOnth, day, and almost every hour throughout
the year, in a great variety of places chiefly in Europe. The more we ex-
amine these tables, the more we admire the indefatigable assiduity of the
authotwho compiled them. These tables are, in reality, worth more thun
double, the price of the book, and deserve a place in every medical library.
We can only make room for the first five columns of a single table, as a
specimen of the whole.
94 Medico-cjiirurgical Review. [Nov.
TABLE
Shewing the mean Annual Temperature of different Localities, and also the
mean Temperature of the Four Seasons.
NAMES OF THE PLACES.
Mean
Annual
Temp.
Mean Temp, of the Seasons.
Winter. Spring. Sumr. Autum
London, 1. (a.)
Edinburgh, 2. (a.)
Leith, 3, (a.)
Kinfauns, 4 *
Dublin, 5. (d.)
County of Antrim, 6
Kendal, 7
Alderley, (Cheshire,) 8. (a.)
New Malton, (Yorkshire,) 9.
Oxford, 10
Environs of London, 11. (a.)
Bushey Heath, 12
Chichester, 12. (a.)
Gosport, 14
Isle of Wight, 15
Cheltenham, 16
Sidmouth, 17
Helston, (Cornwall,) 18
Penzance, 19. (a.)
Geneva, 20. (a.)
Paris, 21. (a.)
Nantes, 22
Bordeaux, 23
Pau, 24
Montpelier, 25.
Avignon, 26. (a.) .
Marseilles, 27. (a.)
Toulon, 28
Nice, 29. (a.)
Genoa, 30
Baths of Lucca, 31
Camajore, (Lucca,) 32. (a.)
Sienna, 33
Florence, 34
Leghorn, 35. (a.)
Pisa, 36
Rome, 37. (a.)
Naples, 38. (a.)
Mediterranean, gen. temp, of, 38. (b.)
Cadiz, 39
St. Michaels, (Azores,) 39. (c.) ....
Madeira, 40. (a.)
Santa Cruz, (Canary Isles,) 41
Cairo, 42

				

